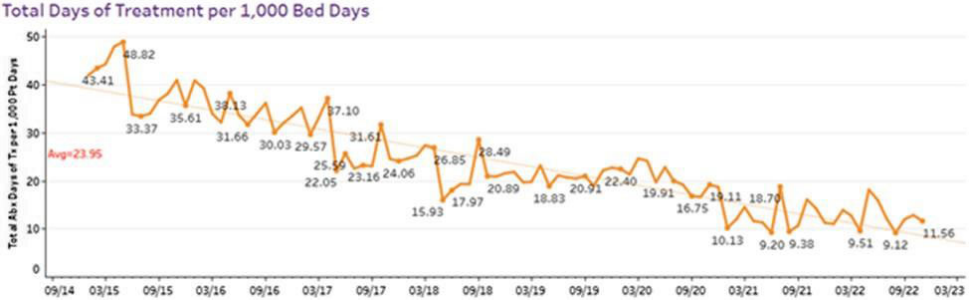# Antimicrobial Stewardship in a Psychiatric Hospital: Opportunities for Improvement

**DOI:** 10.1017/ash.2023.440

**Published:** 2023-09-29

**Authors:** Melissa Rozdilsky, Chloe Bryson-Cahn, Jeannie Chan, Rupali Jain, John Lynch, Natalia Martinez-Paz, Zahra Kassamali Escobar

## Abstract

**Background:** Western State Hospital (WSH) is an 800-bed, state-owned psychiatric hospital in Washington State which services individuals in 20 counties. WSH provides services and inpatient treatment to patients referred via behavioral health providers and/or the civil court system. Because many patients are admitted with serious, long-term illness, WSH also provides primary care and addresses infectious syndromes encountered in admitted patients. In January 2016, WSH officially began their antimicrobial stewardship program (ASP). In 2017 WSH joined the UW Center for Stewardship in Medicine (UW-CSiM) to grow and optimize their ASP. **Methods:** The lead pharmacist at WSH participated in weekly hour-long education and tele-mentoring sessions through the UW-CSiM program. Educational materials were adapted from UW-CSiM didactics and delivered to providers during regular meetings and grand rounds. Daily pharmacist led prospective audit with feedback was conducted. Antibiotic use data were collected and measured by days of therapy (DOT) per 1000-patient days from pharmacy dispensing records from 2015 to 2022. **Results:** From 1/1/15 to 12/31/22, there was a consistent trend of decreasing antibiotic consumption annually. In particular, antibiotic use decreased by over 65% ranging from 35-43 DOT per 1000 patient-days in 2015 to 9-11 DOT per 1000 patient-days in 2022 (Figure 1). This translates to approximately 1000 antibiotic days of therapy in 2015 and 200 days of antibiotic therapy in 2022. As of 2022, the two most common antibiotics used were cephalexin and sulfamethoxazole/trimethoprim **Conclusion:** Although treating infections is not a principal focus of a psychiatric hospital, patients receiving care in inpatient psychiatric facilities do experience common infections and receive antibiotics during their stay. At WSH, initiation of an antimicrobial stewardship program was associated with sustained decrease in total antibiotic DOT over 7 years. These data highlight the impact of tele-education and tele-mentoring in infectious diseases and antimicrobial stewardship as a path to build a successful antimicrobial stewardship even without formal infectious diseases training. Our single center experience at a large psychiatric hospital demonstrates the use of antimicrobials in these facilities and the opportunity for a large impact of an antimicrobial stewardship program in inpatient psychiatric facilities.

**Disclosures:** None.

**Figure:**